# Aping Language: Historical Perspectives on the Quest for Semantics, Syntax, and Other Rarefied Properties of Human Language in the Communication of Primates and Other Animals

**DOI:** 10.3389/fpsyg.2021.675172

**Published:** 2021-07-23

**Authors:** Drew Rendall

**Affiliations:** Department of Biology, University of New Brunswick, Fredericton, NB, Canada

**Keywords:** language, evolution, animal communication, primates, semantics

## Abstract

In 1980, Robert Seyfarth, Dorothy Cheney and Peter Marler published a landmark paper in *Science* claiming language-like semantic communication in the alarm calls of vervet monkeys. This article and the career research program it spawned for its authors catalyzed countless other studies searching for semantics, and then also syntax and other rarefied properties of language, in the communication systems of non-human primates and other animals. It also helped bolster a parallel tradition of teaching symbolism and syntax in artificial language systems to great apes. Although the search for language rudiments in the communications of primates long predates the vervet alarm call story, it is difficult to overstate the impact of the vervet research, for it fueled field and laboratory research programs for several generations of primatologists and kept busy an equal number of philosophers, linguists, and cognitive scientists debating possible implications for the origins and evolution of language and other vaunted elements of the human condition. Now 40-years on, the original vervet alarm call findings have been revised and claims of semanticity recanted; while other evidence for semantics and syntax in the natural communications of non-humans is sparse and weak. Ultimately, we are forced to conclude that there are simply few substantive precedents in the natural communications of animals for the high-level informational and representational properties of language, nor its complex syntax. This conclusion does not mean primates cannot be taught some version of these elements of language in artificial language systems – in fact, they can. Nor does it mean there is no continuity between the natural communications of animals and humans that could inform the evolution of language – in fact, there is such continuity. It just does not lie in the specialized semantic and syntactic properties of language. In reviewing these matters, I consider why it is that primates do not evince high-level properties of language in their natural communications but why we so readily accepted that they did or should; and what lessons we might draw from that experience. In the process, I also consider why accounts of human-like characteristics in animals can be so irresistibly appealing.

## The Vervet Alarm Call Story and Its Enduring Legacy

Seyfarth et al. (1980a) published a landmark paper in *Science*, reporting what was interpreted to be evidence for language-like communication in vervet monkeys, a species of primate relatively distantly related to humans. The paper reported that vervet monkeys gave acoustically distinct alarm calls to different types of predator which prompted functionally distinct escape responses in listeners. It was argued that the calls were not simply emotively based and that contextual details were not needed in order for listeners to respond appropriately. Rather, the calls alone were sufficient to elicit the distinct escape responses. Hence, the calls appeared to function as symbolic labels for the predators, much like our human words for them, and were interpreted as the first evidence for semantic communication in a primate. Because the alarm vocalizations showed no iconic resemblance to the predators themselves, they were also claimed to exemplify the property of *arbitrariness* that the linguist Saussure had previously proposed to be a defining structural property of human words (de Saussure, 1971). Here then appeared to be evidence for language-like communication in a non-human primate with the potential also for some similar human-like cognitive abilities. The implications for the evolution of language and mind in humans – topics that had bedeviled scholars for ages – were tantalizing.

Indeed, the impact of the 1980 *Science* paper was profound. Although its findings were never replicated (until they couldn’t be: Price, 2013; Price et al., 2015), the paper nevertheless became the textbook example of language-like communication in animals and catalyzed a successful career research program for its primary authors, Seyfarth and Cheney focused on other evidence of human-like behavior and cognitive abilities in primates. Much of that research program was summarized for a wider audience in two successful popular books entitled, *How Monkeys See the World: Inside the Mind of Another Species (1990a)*, and *Baboon Metaphysics (2008)*, the latter title a nod to Darwin who suggested that philosophers of mind at the time, like Locke and others, would get more traction on the problem of human psychology (metaphysics) by studying baboons (as Seyfarth and Cheney indeed did: Cheney and Seyfarth, 2008).

The vervet alarm call story also catalyzed countless other studies searching for rudiments of semantics, and then also syntax and other rarefied properties of language, in the communication systems of other primates and a variety of non-primate species besides. And it served also to bolster a parallel historical tradition attempting to teach symbolism and syntax in artificial language systems to great apes.

In fact, it is difficult to overstate the impact of the original vervet alarm call story, for it helped to fuel field and laboratory research programs for several generations of primatologists, right up to the present, and kept busy a significant number of philosophers, linguists, and cognitive scientists debating possible implications for the origins and evolution of language and other vaunted elements of the human condition (e.g., Dennett, 1983; Premack, 1985; Bickerton, 1992; Pinker, 1994; Deacon, 1998; Hauser et al., 2002; Pinker and Jackendoff, 2005; Fitch, 2010; Berwick and Chomsky, 2016).

Now 40-years on, the original claims for semanticity in vervet alarm calls have recently been recanted (Price, 2013; Price et al., 2015; Seyfarth and Cheney, 2017) and additional evidence for symbolism, syntax, or other high-level intentional and informational properties of human language in the natural communications of non-humans is thin (Wheeler and Fischer, 2012; Scott-Phillips, 2015; Fischer and Price, 2017).

In hindsight, these outcomes might have been anticipated given other standard features of communication in non-human primates, including: that they have relatively small repertoires of different calls and use them in a wide range of contexts with little context-specific usage suggestive of discrete messages; that most calls are graded variants on a few basic structural themes of coos, grunts, barks and screams; that there is little cortical control of vocal production which is instead largely limbically driven and closely tied to emotions; that there is a conspicuous absence of social-cognitive intentionality in communication or other aspects of their behavior; and that there is little evidence of productive vocal learning; all of which are hallmarks of human speech and language (reviewed in Owren and Rendall, 2001; Penn and Povinelli, 2007; Hammerschmidt and Fischer, 2008; Jürgens, 2008; Hage, 2010; Owren et al., 2010; Rendall and Owren, 2013; Fischer and Price, 2017; Nieder and Mooney, 2020; Fischer, 2021).

Ultimately, we are forced to conclude that, although there may be some superficial resemblances, there are simply few substantive precedents in primates, or other species, for the high-level intentional, informational and representational properties of language, nor its complex syntax.

This conclusion was, in fact, reached by Cheney and Seyfarth (1998, 2005) themselves some time ago (1998, 2005) with their conclusion that primate communication is fundamentally not intentional the way language is: “*non-human primates’ inability to represent the mental states of others makes their communication fundamentally different from human language*” (Cheney and Seyfarth, 2005, p. 135). These conclusions have been echoed and extended by other prominent researchers in the field. For example, Marc Hauser, formerly a notable figure in this line of research, concluded that: “*Although 40 years of research have been invested in the capacity of animals to produce or comprehend externalized symbols, the relevant evidence that they do so is, at best, weak*” (Hauser, 2009, p. 194). In a subsequent review of the state of research on language evolution, Hauser et al. (2014) later concluded that, “*Animal communication systems have thus far failed to demonstrate anything remotely like our systems of phonology, semantics, and syntax.*” Michael Tomasello, in his book, *Origins of Human Communication*, concluded that: “*Primate vocal displays are basically no different from those of other mammals. Vocal displays are mostly unlearned, genetically fixed, emotionally urgent, involuntary, inflexible responses*… *How could such mechanical reflexes be a direct precursor to any of the complexities of human communication and language*…*?*” (Tomasello, 2008, p. 53).

Revealingly, these conclusions have done little to slow the pace of research in this area. To wit, at the close of 2020 (and according to Google Scholar), the original 1980 *Science* paper on vervet alarm calls had been cited almost 1,500 times, and a companion paper published the same year in the journal *Animal Behavior*, also under the banner of semantic communication (Seyfarth et al., 1980b), had been cited just over 1,000 times – with no sign that the rate of citation of either paper has slowed since most of the above-noted conclusions were reached. On the contrary, the rate of citation for both papers is actually higher post-2000 compared to before. The authors’ two popular books are even more widely cited. In early 2021, *How Monkeys See the World* had been cited 3,795 times and *Baboon Metaphysics*, published only in 2008, has already been cited 974 times. When one considers that the thousands of researchers who have cited these various works have themselves likely also been cited by hundreds, possibly thousands, of other researchers – a calculus of spread now familiar to us all in the midst of the global COVID-19 pandemic – it is obvious just how widely impactful the vervet alarm call story has been. Clearly, its appeal was and remains strong, and its influence has spread broadly and deeply, such that it could be some time before news of its revision reaches the diverse literatures where it has taken hold.

Note that although the weight of evidence now does not support the conclusions of the original *Science* paper, nor most other language properties it spurred the study of in other species, this outcome does not in any way represent a critique of the intelligence, achievements or inherent worth of any of the species studied in the process. This is a very important point that I’ll return to later. Likewise, the conclusion also does not mean that there is no constructive continuity between primate and human communication – in fact there is continuity and it includes: some similar elements of basic vocal anatomy and basic processes of vocal production and thus similarity in the resulting sounds produced; some similarity in the peripheral mechanisms of vocal perception; some flexibility in call production and usage; a role for feedback in shaping infants’ vocal production development; and a role for facial and other gestures in complementing vocal communication. Together these areas of overlap may point to some basic common building blocks of communication that could be part of the scaffolding for human communication, ultimately including speech and language, even if they do not yet illuminate much about the emergence of higher-level properties of language, such as its semantics and syntax. There are many constructive reviews of this evidence with suggestions for where future research could productively focus (e.g., Rendall et al., 1998, 2004, 2005; Fitch, 2000, 2020; Davila Ross et al., 2008, 2010; Ghazanfar and Rendall, 2008; Takahashi et al., 2015; Griebel et al., 2016; Boë et al., 2017; Nielsen and Rendall, 2018; Pomberger et al., 2018; Ghazanfar et al., 2019; Oller et al., 2019; Dezecache et al., 2020; Locke, 2021 this issue). Hence, I will not dwell further on that evidence here to rehearse what is well covered elsewhere.

Instead, I will focus in this article on two other broad and important questions that are prompted by the enduring legacy of the vervet alarm call story but that have never before been asked or addressed: **First, why are core properties of language, in fact, not manifest in the natural communications of non-human primates? And second, why did we ever think they should be?**

## Why Are Semantics and Syntax Not Manifest in Primate Communication?

### The Role of Intentionality

On the one hand, the answer to this question is quite straightforward, because there is a natural organizational hierarchy inherent in the semantic and syntactic properties of language that is grounded in its underlying intentionality. Thus, in human language, canonical speech acts are predicated on implicit (and sometimes explicit) mental state attributions about our audience, namely that they have thoughts or beliefs that are *about* the world that we want to engage by communicating with them (Grice, 1957). For example, they might think X, and we’d like to affirm for them our own understanding of X, or change theirs. Or we may think that they do NOT have any such knowledge about X but should. And so we tell them about X. Either way, our capacity for thoughts that are *about* things and for attributing the same capacity to others – to viewing them, like ourselves, as mental agents with internal states of knowledge or belief that are also about things – is referred to formally as *intentionality* (sensu Brentano, 1874). More prosaically, intentionality represents the cognitive impetus *to inform* others (or affirm, change, influence, or otherwise engage, their mental states). Of course, this informing function of language is only part of how language works. Nevertheless, it is the essential foundation for the canonical semantic and syntactic properties of language which have been the focus of parallel comparative research on animals, where semantics represents the conceptual content of all that informing, and syntax represents the higher-order organizational rules that emerge with a need to organize more complex semantics.

For most (possibly all) non-human primates, formal intentionality – mental state attribution – appears to be lacking (Cheney and Seyfarth, 1998, 2005; Penn and Povinelli, 2007; Call and Tomasello, 2008). Chimpanzees appear to understand the goal or purpose of another individual’s behavior in instrumental terms – at least in controlled settings interacting with human partners – but they do not appreciate the mental states that lie behind others’ behavior and how that affects what they will do, nor do they act deliberately to alter those mental states (Call and Tomasello, 2008). Studies of other primates have confirmed a similar lack of appreciation for others’ perspectives or mental states, including specifically in communication. For example, Cheney and Seyfarth (1990b) explained early on that, while a vervet monkey will produce alarm calls when it perceives itself to be in danger, that same individual fails to call on other occasions when it is not in danger itself even though other companions, including kin, are at risk. Cheney and Seyfarth (1990a) subsequently replicated this finding with Japanese and rhesus macaques in a series of controlled experiments in captivity designed specifically to systematically test intentionality in communication. In that work, they found that mothers likewise failed to warn their infants of an imminent danger if the situation did not also represent a threat to the mother herself, and they concluded that this was because mothers failed to appreciate the perspective and knowledge of their infants when it differed from their own. Similarly, in field experiments on baboons, Cheney et al. (1996) showed that adult females routinely produce very loud bark vocalizations – informally termed “lost calls” – when they get separated from companions during daily travels in an effort to re-establish contact with the group. However, those same females do not respond vocally to the “lost calls” of other group members who become separated and are trying to relocate the group if they themselves are now safely with the rest of the group at the time. This failure to vocalize to inform others of the group’s location held true even when the separated and calling group member was a female baboon’s own young infant (Rendall et al., 2000).

Taken together, the corpus of work on primate intentionality consistently shows that the animals often attend carefully to the behavior of others, but fail to appreciate what lies behind it. They fail to appreciate others as mental agents with perspectives and resulting states of belief or knowledge that can differ from their own and that in turn affect their behavior. Hence, they fail also then to appreciate how their own behavior, including their own vocalizations, might serve to inform others – i.e., to change their states of belief or knowledge, and thus also their behavior. Hence, they vocalize when they themselves encounter a predator, find food, or become lost but this calling is entirely self-centered, reflecting their own current situation and needs. It does not reflect the informational needs of receivers. In contrast, human language is fundamentally “other-centered” in being routinely tailored to the perceived informational needs of listeners (Owren and Rendall, 2001).

Ultimately then, where formal intentionality in communication is lacking, there is simply no need or capacity for semantics or syntax. In other words, lacking the fundamental underlying cognitive impetus to inform others based on an appreciation of their states of knowledge or belief about the world, there is no functional need in non-human primates for any conceptual informing content to begin with, and therefore also no need for a higher-order syntactic system to organize more complex messages.

It is worth noting here that, in view of the negative findings on primate intentionality, an alternative conception of primate semantics arose in the 1990’s that was referred to as “functional reference” and promoted continued work on the subject (Marler et al., 1992; Macedonia and Evans, 1993; Evans, 1997). The proposal was that, although primates were evidently not vocalizing intentionally to inform others about things in the world, as is the case for routine human language use, their vocalizations might nevertheless function “as if” they were. For example, while a vervet monkey producing an alarm call might do so with respect to its own circumstances and its own associated concern, fear or distress on encountering a predator, as is now accepted to be the case (Price et al., 2015; Seyfarth and Cheney, 2017), other group members hearing the call might still respond appropriately “as if” the call had conveyed semantic information about the predator type. In the original formulation of the framework, the threshold for assigning functional reference was that the vocalization elicited appropriate responses from listeners in the absence of supporting contextual information (Macedonia and Evans, 1993). By this criterion, vervet alarm calls would now not qualify even for this looser characterization of reference, given that listeners are now acknowledged to require additional contextual details to respond appropriately (Price, 2013; Price et al., 2015; Seyfarth and Cheney, 2017).

Latterly, however, this context-free criterion seems to have been relaxed further to allow signals, such as vervet alarm calls, to be labeled functionally referential if listeners can respond appropriately using additional information available from the immediate contextual details as well as what they have learned from past experience about the kinds of events that are associated with specific vocalizations from companions (Seyfarth and Cheney, 2017). In this way, what could be largely affective or motivationally driven vocalizations in signalers might nevertheless be interpreted to retain an element of external reference by virtue of additional inferences listeners make based on other information they glean from the current situation or past experience. On the surface of it, this is a perfectly sensible parsing of how the monkeys actually behave and respond. After all, primates are large-brained animals with significant inferential capacities. So, almost certainly, they routinely respond to vocalizations from companions based on a variety of inferences they make using a combination of immediate contextual details, their familiarity with group members and their individual behavioral proclivities, and their familiarity also with the circumstances that typically elicit different kinds of signals from them (cf. Smith, 1977; Owings and Morton, 1998).

At the same time, however, if vervet alarm calls function in this manner then they are effectively no different than any of the other vocalizations in their repertoire, or in the vocal repertoire of any other species, such as the common grunts, barks, squeals and screams that mediate quotidian activities. All of these calls too are largely affectively driven and reflect the current situation and needs of the signaler but could nevertheless similarly allow listeners to draw additional inferences about likely eliciting circumstances based on additional contextual information and familiarity with each other and their general proclivities.

Indeed, such a parsing of the function of vervet alarm calls simply aligns them with a wide range of other signaling phenomena not typically considered language-like at all, including, for example, human infant crying (and “crying” in other species). While the crying of human infants is definitively emotionally driven, parents can nevertheless often infer some general things about the eliciting circumstances, such as whether the cries reflect being overtired, or hungry, or in pain or general distress based on familiarity with their own infant, its crying patterns, and other contextual details including the time since last feeding, or the infant’s recent sleep history (reviewed in Zeifman, 2001; Soltis, 2004). As a result, there does not appear to be any explanatory value added by attaching the label “reference” to vervet alarm calls, or any of these other signals, in order to promote comparison to language if the label could be applied equally well to all of these other common signals that are so clearly not language-like at all.

These points were well appreciated by earlier key researchers (see Premack, 1972; Marler, 1977; Owings, 1994), who therefore recognized an important distinction between such signaler-centered, affectively based vocalizations (in animals or humans) and the truly referential or symbolic quality of human language. The key distinction, as noted earlier, is that linguistic reference hinges on the intentionality of language users. That is what moves language beyond being exclusively sender-centered to being also receiver-centered, because language acts are routinely conditioned not only by the immediate circumstances of the speaker but also, and specifically, the informational needs of listeners. It is also what confers the representational power of language, allowing it to move beyond contextually bound signals that can be interpreted only with additional details of the immediate circumstances, to the context-free and virtually unbounded representational universe of human language instantiated by words that have common representational value for signaler and receiver alike and are understood by both parties to have such representational value.

In short, without intentionality, communicative acts have no meaning in the formal linguistic sense, and cannot be scaffolded into more complex semantic constructs that create pressure for organizational systems (grammars) to organize them. So it is precisely the psychological characteristic of formal intentionality explained earlier that kicks off the complexity of linguistic reference and that an evolutionary account of language therefore needs to account for. It is, therefore, illusory to search among primates, or other animals, for vocalizations that are referential only indirectly in some functional and not intentional sense, maintaining that this will somehow provide any illumination on the evolution of linguistic reference (reviewed in Rendall et al., 2009; Owren et al., 2010; Rendall and Owren, 2013).

So, while understandable enough as a conceptual retreat regarding primate semantics in response to mounting evidence for a lack of language-like intentionality in their communication, the functional reference gambit actually muddies comparisons between language and primate communication and obfuscates more than it illuminates meaningful points of similarity and difference between them that could ultimately clarify our understanding of the course of language evolution. In a recent comprehensive review of the concept of functional reference, Wheeler and Fischer (2012) drew much the same conclusion, allowing that the functional reference framework was “a promising paradigm whose time has passed.”

In summary then, the straightforward answer to why non-human primates lack semantics and syntax is that they lack the functional prerequisites to each: they lack syntax because they lack the prerequisite complex semantics, and they lack semantics because they lack the prerequisite intentionality. There is a functional hierarchy to these properties of language, where, for non-human primates, the ground floor is missing.

It is very important to appreciate the contingent functional nature of these language properties, and why they are then absent in non-human primates. At the same time, however, this understanding seems just to push the matter back one step. **Why would these canonical features of language not also be important to non-human primate communication?**

### The Natural Environment of Primate Communication

That’s a different and important question – the “other hand” of the issue as noted above. Currently, it’s impossible to say definitively why primates do not manifest semantics or syntax in their natural communications because the question has never been explicitly posed and studied in that way. However, traction on the question is likely to come from refocusing on the natural history, environment and behavior of the animals themselves – rather than seeing them as stand-ins for human ancestors – and assessing how their communication in fact serves the needs of their world, rather than ours. In this, there is a wealth of relevant literature to draw on from the significant bodies of research on primate behavior and ecology generally, and on general aspects of their communication unrelated to the question of language. And, while Primates are a large and diverse Order, there are some common elements that stand-out in these literatures and provide fertile ground for addressing the question (Smuts et al., 1988; Mitani et al., 2012).

Very briefly then, many primate species are highly social, even those that might typically be labeled as “solitary” because they do not live in permanent groups. There are important exceptions, of course, but to the extent broad generalization is possible, and despite many variations in other details of their behavior and ecology, primates are generally held to occupy a distinctly social niche. Many species live in stable groups, or looser communities, comprised of individuals of varying age, sex, social rank, and degrees of relatedness. And most species are also relatively long-lived. Hence, there is protracted opportunity for development of a complex web of differentiated social relationships among group members according to these various social distinctions. Indeed, the conclusion from many decades of research is that these different social relationships powerfully affect all manner of daily activities and have resulted in a highly developed “social acumen” (Jolly, 1966; Humphrey, 1976; Dunbar, 1998).

Many daily activities are also mediated by vocalizations, including soft coos and grunts that mediate relatively relaxed, affiliative social contexts; loud barks and screams that mediate aggressive conflict; and excited squeals and shrieks in the context of food discovery or predators. Many of these contexts have a particular immediacy to them and are strongly valanced, either positively or negatively, with variation in the intensity thereof. And, while innately given, the vocalizations produced in these contexts also reflect that variable valencing of events through marked grading in the amplitude, tempo and spectral structure of the calls in any particular circumstance. Vocalizations produced in some of these contexts also manifest structural differences attributable to a variety of indexical dimensions, namely differences in the age, sex, size and often also individual identity of the caller. Hence, the social attunement of the animals and their rich and differentiated social histories provides broad scope for inference and interpretation of such affectively laden signals – as noted earlier – according to the identity of the caller and their age, sex, rank, and kinship relative to listeners; further conditioned by the recent and longer-term nature of their relationships to one another; and by myriad elements of the immediate behavioral and environmental context associated with calling. Taken together, this mix of cues from available contextual details, the social identity of signalers, and the dynamic social history of participants describes a pretty rich platform for flexible and functional communication in support of a host of quotidian social and behavioral routines. But not one where there is an obvious selective need for anything like the semantic or syntactic properties of language.

This is a necessarily truncated parsing of primate communication for present purposes and is not intended to decide the question of why primates do not evince semantics or syntax but rather only to open it. Nevertheless, it is a parsing that aligns with many earlier proposals (Smith, 1977; Krebs and Dawkins, 1984; Premack, 1985; Leger, 1993; Owings, 1994; Owings and Morton, 1998; Owren and Rendall, 2001; Wheeler and Fischer, 2012) and also with very recent and comprehensive reviews of the subject (Fischer and Price, 2017; Fischer, 2021). Notably, it is a conclusion endorsed recently also by Seyfarth and Cheney (2017). In revisiting the original vervet alarm call work in the light of their own recent revised findings, they conclude together with colleagues that: “*We suggest that both cognitive appraisal of the situation and internal state contribute to the variation in call usage and structure. While the semantic properties of vervet alarm calls bear little resemblance to human words, the existing acoustic variation, possibly together with additional contextual information, allows listeners to select appropriate responses”* (Price et al., 2015, page 1). And in a more recent broader review of the topic, Seyfarth and Cheney (2017) now emphasize the constrained nature of call production in primates that is largely innately given and tied importantly to affective motivations, making contextual details important to listeners in interpreting and responding to the vocalizations of others in any given situation. These recent acknowledgments represent a pretty significant reversal of perspective from having originally and explicitly discounted the importance of affect and contextual details in vervet alarm calls in favor of the semantic quality of the calls alone based on categorically distinct acoustic structures and specific contexts of usage (Seyfarth et al., 1980a). Notably now, however, they reinterpret the above-noted characteristics of primate communication under the banner of linguistic *pragmatics* rather than *semantics.* So, with this revised perspective, they continue to attempt concrete connections to language even while acknowledging that the previous focus on semantics was misplaced. **Which prompts again the question, why we ever expected primate communication to evince semantic or syntactic properties similar to human language to begin with?**

## Why Did We Ever Think Non-Human Primate Communication Should Show Language-Like Semantics or Syntax to Begin With?

On the one hand, it is a perfectly sensible intuition that primates might manifest some of the rudiments of human communication, possibly including language. They are, after all, our closest living relatives. Hence, it is entirely reasonable to study primate communication for elements of it that might inform our understanding of the origins and evolution of language. On the other hand, it might feel a backward, or at least a bit strained, to search first specifically for evidence of the high-level intentional and representational properties of language, such as its semantics and syntax, if these are at all likely to be relatively recent, derived and specialized properties of communication in humans. In which case, it might feel strained to expect meaningful precursors of such specialized language properties in species so far removed from modern humans. It may even feel logically backward to effectively project such derived properties of modern language backward in time to the communication systems of living species as stand-ins for human ancestors assuming these properties of language must also be present and functional for them as well, even if in more rudimentary form (Premack, 1985; Pinker, 1994; Rendall et al., 2009).

Whatever one’s stance, it should be noted that scholarly and popular interest in the possible evolutionary precursors to language in primate communication long predates the vervet alarm call story. Up until that time, however, research on the subject was relatively spotty and fragmented. **So, the pertinent issue for present purposes is really why the vervet alarm story was so especially impactful and how it served to consolidate a much more focused and enduring research agenda?**

This is an important and potentially multi-faceted question. Part of the answer may lie in effects attributable to the historical, intellectual climate of the time – and a paradigm shift in that – as well as to the specific intellectual commitments of key players involved in that paradigm shift. Another part may involve broader influences on the wider audience that affected their reception to, and uptake of, the original findings.

## What Motivated the Focused Search for Semantics and Syntax That Accompanied the Vervet Alarm Call Story?

### The Rise of Cognitivism

One can trace the roots of this focused search to a couple of parallel scientific developments of the mid-20th century. The first of these was the rise of cognitivism in Psychology which involved shedding the shackles of Behaviorism. Importantly, this paradigm shift included a specific focus on language and also included a very public showdown between Chomsky and Skinner concerning the extent to which behaviorism or cognitivism represented the better approach for understanding human language (and behavior more broadly). Chomsky was the decided winner and, while certainly not alone in this, was a central figure in helping to usher in a cognitive revolution that took hold in Psychology and ultimately transformed many disciplines, and even invented some entirely new ones (e.g., Cognitive Science) all with a fresh focus on human mental experience. Many researchers in animal behavior were also quick to embrace cognitivism. After decades of the strictures of Behaviorism in the study of animals as well, they too were poised to think again about animal mental life. This focus ultimately led to a reorientation and rebranding of a whole branch of animal behavior research under the banner of Cognitive Ethology.

A second important and complementary development involved research at about the same time specifically on animal communication. A key early figure in this development was Donald Griffin at Rockefeller University who, together with others, was responsible for solving the mystery of how bats navigate in the dark – namely through production of a continuous stream of high-frequency (ultrasonic) clicks and detection of their reflected echoes off objects in the environment, a process dubbed echolocation. This was an exciting finding that helped to illuminate (for us) the dark world of the bat. For Griffin, it also captured how communication, among all behaviors, could be a privileged source of insight into animal mental experience – a “window into their minds” as he put it (Griffin, 1995). He promulgated this notion for a wider audience in a popular book entitled *The Question of Animal Awareness (1976)*, and another titled, *Animal Thinking (1985)*, in which he wholly re-popularized the formerly taboo subjects of animal mental experience and animal consciousness under the new banner of Cognitive Ethology. In the first of these books, he also explicitly forged the connection to language in a way that would help frame the subsequent conceptual agenda for much comparative research on primates, writing that:

“*In so far as linguists and philosophers have been correct in linking human thinking so closely to language, the communication behavior of other species is bound to suggest conscious thought to roughly the extent it shares essential features with human speech*” (Griffin, 1976: p. 39).

### The Rockefeller Effect

These various threads were tied together by Peter Marler, who was by then a colleague of Griffin’s at Rockefeller, having relocated earlier from Berkeley. Marler’s career research program – prior to this and following – was focused primarily on birdsong, though with an abiding interest in comparisons to language. Significantly at the time, though, he had recently supervised a stand-out graduate student named Tom Struhsaker at Berkeley. Struhsaker had conducted a comprehensive field study of vervet monkeys and published a monograph on his research (Struhsaker, 1967). It focused primarily on the natural history of the monkeys, general dimensions of their behavior and ecology, but it also included a section on communication. Importantly, that section contained preliminary descriptions of a small repertoire of alarm calls produced in reaction to different kinds of predators. Struhsaker did not pursue the matter in detail. However, for Marler, the combination of Griffin’s local influence at Rockefeller and the specific research challenge Griffin had laid down connecting animal language to conscious thought, assuredly left Struhsaker’s brief descriptions of the vervet alarm calls pregnant with possibility.

At Rockefeller then, Marler recruited as postdocs Robert Seyfarth and Dorothy Cheney fresh from completing PhDs on the social behavior of baboons in South Africa, and he dispatched them to Kenya to followup Struhsaker’s preliminary descriptions of vervet alarms. Marler also recruited two other teams to conduct similar studies on other primate species, one of them another husband and wife couple, Harold and Sally Gouzoules, and the other, Steven Green. Notably, all three teams returned reports of categorically distinct vocalizations interpreted to manifest parallels to the semantic properties of language, the first ever such reports from the natural communications of primates.

Seyfarth and Cheney returned the now familiar vervet alarm call story (Seyfarth et al., 1980a). Further details of their fieldwork and how they came to their conclusions is treated thoroughly in an engaging book by the historian of science, Gregory Radick (Radick, 2007). The Gouzoules’ studied loud scream vocalizations given by many primate species when physically attacked by social companions. They returned evidence of distinct variants of scream in rhesus monkeys that were proposed to convey a host of representational information to listeners about the severity of the aggression involved, the social rank of the attacker, and the degree of kinship between attacker and victim (Gouzoules et al., 1984). And Steven Green studied “coo” vocalizations produced by Japanese monkeys in a variety of social contexts and provided a typology of different kinds of coo which were reported to be perceived and interpreted categorically much like the sounds of human speech (Green, 1975; Zoloth and Green, 1979). Neither of the latter two studies garnered quite the same attention as the vervet alarm call story, nor were they ever replicated, but collectively they helped catalyze an enduring tradition of language parallels research in primates and beyond.

Much of this enduring tradition of language parallels research, particularly that focused on primates, continued to be closely connected to Marler’s trainees, such as Seyfarth and Cheney, and then their trainees, and the trainees of those trainees in turn, each focused on additional evidence of semantics, and then also syntax, in the vocalizations of primates (e.g., Gouzoules et al., 1984; Evans et al., 1993; Macedonia and Evans, 1993; Hauser, 1998; Zuberbühler et al., 1999; Manser et al., 2002; Slocombe and Zuberbühler, 2007; Arnold and Zuberbühler, 2008; Crockford et al., 2015; Zuberbühler, 2019). So, there is a very close connection of this research tradition to a family of researchers emanating from Marler and Rockefeller University.

At the same time, the research tradition forged by this group has also spread much more widely and ultimately taken in a wide range of other researchers and a broad array of animal species from chickens and chickadees, to meerkats and marmosets, to prairie dogs and squirrels (e.g., Evans et al., 1993; Greene and Meagher, 1998; Manser et al., 2002; Templeton et al., 2005; Kitzmann and Caine, 2009; Slobodchikoff et al., 2009). It is important to ask then what accounts for this wider appeal? How did this line of research captivate such a broad audience?

## Why Was the Wider Audience So Receptive to the Vervet Alarm Call Story?

The citation statistics for the vervet alarm call story noted earlier are striking, particularly when one appreciates that those citations represent only the record of formal published works explicitly influenced by the original study. The more informal influence of the original story on scholarly thought and research programs must be far more extensive.

This broad influence is the more remarkable when one considers that the original work was only a single, unreplicated study. And more remarkable still when one appreciates that the original evidence was also quite mixed. As reported in the original papers, the alarm calls were not, in fact, given exclusively to predators but were used also in other contexts characterized by high arousal such as within group aggression; there were acoustic gradations among the various calls; and the monkeys’ responses to hearing different alarm variants were also mixed. Indeed, only some of the main effects tested experimentally in the original *Science* paper were statistically significant and then only very few using a conservative alpha level of *0.01*. The other effects interpreted as significant were subject to relaxed alpha levels between *0.05* and *0.10*. So, the production and response patterns for the different alarm calls were never either definitive nor exclusive.

This mixed pattern of call usage in both alarm and non-alarm contexts, and the overlap in call structures between the proposed alarm variants, has been quantified and confirmed much more thoroughly in a recent study deliberately revisiting the original vervet study (Price, 2013; Price et al., 2015) leading to the revised characterization of the alarm calls quoted earlier:

“*We suggest that both cognitive appraisal of the situation and internal state contribute to the variation in call usage and structure. While the semantic properties of vervet alarm calls bear little resemblance to human words, the existing acoustic variation, possibly together with additional contextual information, allows listeners to select appropriate responses”* (Price et al., 2015, page 1).

However, this variability and interpretation was not emphasized nor widely appreciated at the time of the original study, nor since. Somehow the subtleties got lost in the re-telling of the work which came to emphasize the discrete, categorical nature of the calls to pick out different types of predator and thus their apparent language-like semantic properties. In fact, given that citations of the authors’ two popular books exceed those of the original primary publications by a factor of 3:1, it is possible that many scholars who have cited the vervet work are not actually familiar with the original paper and its findings.

This is all the more remarkable given that the implicit, if not explicit, scholarly code in science is that – *extraordinary claims require extraordinary evidence*. This well-known prescription is originally attributed to the French polymath, Laplace, and was popularized in contemporary times by the cosmologist, Carl Sagan (Gillispie, 2000). It is a scholarly code well-known to researchers in evolutionary-oriented disciplines from the writings of George C. Williams, a luminary in evolutionary biology in the 20th century. Williams invoked the prescription in reference to claims of adaptation (e.g., Williams, 1966). He regarded adaptation as a weighty construct and indeed one that had to be if it were to have any real value in evolutionary theory (cf. S. J. Gould). Hence, he argued that claims of adaptation cannot be made lightly, nor accepted uncritically by others. Instead, claims of adaptation bear a heavy burden of proof. Researchers advancing claims of adaptation and readers evaluating them must be equally circumspect and dually committed to high standards of evidence.

Although Williams helped to codify this notion in evolutionary biology in the mid-20th century, it was appreciated by evolutionists well before that. Darwin himself might be the paradigm example. He had to be literally cajoled to publish his theory of evolution by natural selection and he took the better part of 30-years to muse on it and to amass the requisite evidence before releasing it in print. Darwin knew his “dangerous idea” (cf. Dennett, 1995) would seem an extraordinary claim; hence, it needed extraordinary evidence, and he took 30-years to carefully and comprehensively accumulate it. Darwin’s diligence delivered significant dividends. Most of his core insights, some very far-reaching, have stood the test of time.

There feels like a lesson here for contemporary science, now conducted at break-neck pace by comparison and increasingly handmaiden to a host of additional factors quite peripheral to the science itself, including grant funding and prestigious awards, impact factors, citation statistics, media coverage, popular attention, and ultimately career advancement.

In another example from the vervet research, Seyfarth and Cheney reported an additional landmark finding in 1984 shortly after the alarm call study was published, this time in the journal *Nature* (Seyfarth and Cheney, 1984). The *Nature* paper reported evidence of tit-for-tat reciprocal altruism in the vervets, a form of cooperation virtually undocumented in animals at that time but thought to be central to the complexity of human social behavior and cooperation. The vervet reciprocity study was based on a sample of nine subjects, which is the minimum sample size required to achieve a significant effect by the non-parametric Wilcoxon test when one of the subjects responds counter to the hypothesis, as was the case. Another extraordinary claim based on thin evidence, now cited more than 600 times. This additional example might appear to be focusing on the vervet research, in particular, but that is not the point. There are likely myriad other similar examples in this and other fields. No doubt any reader of this can point to one or more similar studies in their own particular area of research. And *that* is the point, that some findings have a powerful appeal, absent the usual standard of evidence expected. So what accounts for that? **Why, despite the cautionary prescription about standards of evidence echoed across time by leaders like Laplace, Williams and Sagan, are we so credulous of certain findings? In particular, why are we so credulous of reports of human-like characteristics in animals, such as reports of semantic communication or reciprocal altruism?**

### Our Anthropomorphic Instinct

Possibly because they appeal to our anthropomorphic instinct, our habit of attributing human-like qualities to animals and even to non-material entities (e.g., gods, spirits, the weather, Mother nature, etc.). Non-human primates certainly look like us in many ways, and they can also move and act a lot like us, as well. So, it’s natural to assume that they communicate and think like us, too. Of course, the latter assumption is fraught, particularly when continuity of mental experience serves then as both the *a priori* assumption guiding scientific enquiry as well its conclusion. Nevertheless, the prospect that vervet monkeys, and other species besides, might be a lot more like us than previously known may have been, and continue to be, irresistibly appealing.

Anthropomorphism is a long-standing and possibly universal human practice. Popular and scholarly stance on it has varied. In the middle ages, it was apparently common to put barn-yard animals on trial for bad conduct based on attribution to them of a moral sense (i.e., they should know better). Cartesian dualism subsequently swung the pendulum, proposing that humans were uniquely endowed with the ability to reason and reflect (to cogitate), while animals were driven by emotion and instinct. Animals were likened to mechanical automata, a stance that comfortably distanced us from their instinctual and seemingly brutish habits. And that was part of the cause for distress to Darwin in publishing his major treatise on natural selection: because his theory of evolutionary descent, that would have explicitly connected humans to a shared ancestry with apes and other primates, would surely offend Victorian sensibilities. To wit, the quote often attributed to the wife of the Bishop of Worcester speaking to her husband on hearing Darwin’s idea: “My dear, descended from the apes? Let us hope it is not true. But if it is, let us pray, that it will not become generally known” (Leakey and Lewin, 1977, p. 21).

However, as Darwin’s views did become more widely known and the thinking around evolution and biological continuity became more broadly established in the late 1800’s, the pendulum swung again, back to attributing to animals more sophisticated, human-like forms of reasoning and consciousness, as epitomized in the work of George Romanes. Romanes was a friend and champion of Darwin and enthusiastically extended the biological continuity inherent in Darwinian descent to include continuity of psychological experience as well, in his founding of the new discipline of Comparative Psychology. Romanes was at pains to legitimize study of animal psychology, as a *bona fide* science, which he argued had too long been the purview only of amateurs. In his major treatise (Romanes, 1882: *Animal Intelligence*), he covered a wide range of mental and emotional phenomena across all major animal groups, from invertebrates to primates, inferring mental experience in animals using a combination of personal introspection and analogy. Specifically, he argued that, where the behavior or activity of animals was similar to that of humans, we can infer that the underlying mental operations are also equivalent and are specifically those revealed to us by our own introspection. He was quite explicit in championing this anthropomorphic method. Some of the work involved empirical study, but a lot of it was quite speculative and based on anecdote. And the excesses of this period in comparative study were part of what motivated Lloyd Morgan’s eponymous canon, appealing to the principle of parsimony in application to psychology (i.e., never attribute to the action of a higher mental faculty behavior that can be adequately accommodated by a lower one), and motivated the broader subsequent behavioristic paradigm in comparative psychology in the early 20th Century.

With Thorndike, Watson, Skinner, and others, Behaviorism swung the pendulum once again to a focus explicitly and only on what was concretely observable and measurable – namely behavior – eschewing all reference to internal mental states. The backlash of Behaviorism marked significant progress in empirical methods and techniques for the study of behavior but was quite stultifying in its proscription of all things mental. And, so, by the mid-20th century, in another swing of the pendulum, there was an almost palpable release as the Cognitive Revolution prompted a spirited revival of interest in, and research on, human and animal mental life.

This cognitive revival was closely connected to a growing appreciation of Darwinian evolutionary principles and much greater acknowledgment, both popularly and in scholarly circles, of the connectedness of all living things. The idea of behavioral and cognitive continuity was no longer so threatening as it was in Victorian England when the notion of continuity created considerable dissonance by connecting us to what was assumed to be a brutish animal past. In fact, with attribution of much more human-like characteristics to primates and other animals, Darwinian evolutionary continuity may now be much more flattering in thereby also bequeathing us a more auspicious ancestry. Indeed, modern findings in genetics that reveal clear traces of Neanderthal ancestry in many contemporary human populations (Sankararaman et al., 2014), and evidence for Neanderthals’ more sophisticated material culture (Hardy et al., 2020), have fundamentally changed and improved the popular image of these hominin representatives that were formerly held in very low regard.

Debate about the power versus pitfalls of anthropomorphism is longstanding (Kennedy, 1992; Eddy et al., 1993; Budiansky, 1998; Sober, 1998; de Waal, 1999, 2018; Povinelli et al., 2000; Wynne, 2004; Klopfer, 2005; Barrett et al., 2007; Rendall et al., 2007; Urquiza-Haas and Kotrschal, 2015). Those who support it see tremendous heuristic value and propose that it might even represent an adaptive form of human reasoning – an adaptive human instinct (Barrett, 2005; Urquiza-Haas and Kotrschal, 2015). Attributing human-like traits to fellow humans is obviously wholly natural. It might also be truly adaptive if, as seems plausible, there are fitness advantages to projecting one’s own internal experience onto other humans as a way of “reading their minds” to better understand, anticipate, and influence their motivations and behaviors. Generalizing that strategy of “human projection and mind reading” to other species is also certainly habitual, at least in contemporary western society (cf. Gray, 2020). We routinely talk to our pets and think we understand them, and that they in turn understand us. We also talk to gods and other spirits in the hope that they are listening and will humor us. We see faces in the clouds, and we revel in cartoon animals that are made to talk, act and dress like humans. Indeed, there are whole genres of children’s books, movies and television programs based on deliberately humanized animal characters (e.g., Winnie-the-Pooh; Yogi Bear; Smokey the Bear; Big Bird; Barney; Bagheera and Baloo, Tom and Jerry, O’Malley Cat, Wile E. Coyote, and any of a hundred other Disney and Hanna-Barbera characters). Hence, children in many western cultures, at the least, are enculturated into anthropomorphism, if the habit is not already instinctual. And so whether as adaptive human instinct, or as enculturated habit (or both), attributing human-like characteristics to animals, particularly those most similar to us in other respects such as primates, is common and possibly quite difficult to resist. It is also probably low cost, even if wrong, at least in most of these circumstances … except possibly in scholarly endeavor.

Hence, those who caution against anthropomorphism warn that it is risky to assume commonality of mental experience with other species, the moreso as the phylogenetic distance from humans increases, even where there might be obvious similarity otherwise in external appearance and behavior (Eddy et al., 1993; Povinelli et al., 2000; Wynne, 2004; Barrett et al., 2007). A simple contemporary analogy makes the point: while the Tesla and the gas-powered Jaguar may look a lot alike, have a lot of the same peripheral hardware, and basically do the same functional things (both provide a fast, comfy ride), when you look under the hood, the way they get things done is fundamentally different (a combustion engine versus a really big battery … which isn’t even located under the hood). Critics of anthropomorphism, at least as a scientific methodology, see the same problem viz a viz the internal mental engines “under the hood” that drive our behavior versus that of other species: they might be similar or they could be quite different.

Indeed, extending psychological continuity across broad taxonomic distances glosses a fundamental element of Darwin’s evolutionary insight, namely that the so-called “tree of life” is truly a tree and not a ladder. The latter, ladder-like view of evolution was formerly quite popular, captured in the classic *Scala Naturae*, which envisioned evolution as a linear and progressive process culminating in humans (just short of God). However, that view was rightly abandoned with the Darwinian revolution that emphasized the diversifying effects of the evolutionary process and thus the diversified products it yields. Hence, we now understand species not as rungs on a ladder leading to humans, but rather as the tips of branches of a vast evolutionary tree, where each branch, including ours, describes a distinct path with potentially very distinct evolutionary challenges and solutions. So, it is dangerous to gloss that deep and diversifying evolutionary history and to treat contemporary species – the tips of many different branches – as stand-ins for human ancestors and so as scaled-down versions of ourselves (Hodos and Campbell, 1969, 1990).

This point is well understood by most evolutionary researchers, particularly those who study structure and form (e.g., anatomy) which leaves a fossil record that is tangible and concretely measurable. Here it is possible to trace the distinct evolutionary paths taken by different species through the tangible, measurable evidence of their ancestors. Admittedly, it’s more difficult for researchers interested in mental experience, because mental activity does not fossilize so directly and it is also not so easily accessed or measured even in living descendants (at least not yet). Everything has to be inferred. So, what choice does one have other than to use extant species for comparison to humans? And then how does one go about studying their mental experience? On what do you base your hypotheses of how and what they think? Where is your objective, unbiased point of entry into their mental lives? It’s hard enough to get inside the head of other human beings to truly appreciate their perspectives, thoughts, and feelings; it’s much harder still to get inside the head of another species when their “Umwelt” and resulting “Innenwelt” (von Uexkuüll, 1909) may be so different. Therefore, making the anthropomorphic gambit – projecting our own internal experience onto that of other species – is risky. But what’s the alternative? And so anthropomorphism, a natural human instinct or enculturated habit, practiced routinely and informally in everyday life, elides into a common research strategy in science as well it seems. This elision may often be unconscious or possibly tacit, however, because most scientists would probably deny that they ascribe to anthropomorphism formally and may not even be aware when they are, nevertheless, practicing it.

So, the appeal of the vervet alarm call story, and the broader research program it galvanized, may in part reflect our increasing comfort now with, and indeed the general appeal of, explanations of animals that emphasize their continuity with humans, whether or not we are even aware of our sympathy and appetite for this.

### Conservation and Animal Rights

It’s also possible that some of the appeal of the vervet monkey story, and other work in the genre, reflects the growing movement to engender greater understanding and compassion for other species, an outgrowth of the ever-increasing sense of eco-awareness and conservation that began in the mid-1900’s and has grown steadily since (if too slowly, still), based on a broader understanding of, and commitment to, the inherent connectedness of global ecosystems and their inhabitants.

This sentiment, in more explicit and exaggerated form, finds expression in some of the research conducted on great apes that seeks parallels to language. One of the most celebrated of these concerns the long-running research program by Sue Savage-Rumbaugh with Kanzi, a bonobo (which is a species of chimpanzee, formerly but no longer referred to as the *pygmy* chimpanzee). Kanzi was reared from birth in a language research environment and was regularly immersed in human routines such as cooking meals and camping out in the forest. Ultimately, he learned a large number of artificial symbols. The work also garnered a lot of public attention and press, some good and some bad. It was popularized for a wide audience in a book co-written with the science writer, Roger Lewin, titled, *Kanzi: The Ape at the Brink of the Human Mind* (Savage-Rumbaugh and Lewin, 1994). Here and elsewhere, his tutor and advocate, Sue Savage-Rumbaugh, proposed that Kanzi be extended human rights. She even took the step of adding Kanzi as a co-author on research publications, apparently as *ipso facto* proof of his humanity (Savage-Rumbaugh et al., 2007).

Here, the effort to persuade others to extend human rights to Kanzi effectively turns on his mastery of language skills, the logic being that any creature possessed of sophisticated language abilities must be sentient in a very human-like way and thus must be accorded some of the same rights and privileges (an extension of Don Griffin’s argument connecting language-like communication in animals with conscious thought). Put simply: If language, then human. To be sure, the objective here is laudable – but it might not be objective. Certainly, it must put added pressure on the research program, and Kanzi as pupil, to demonstrate facility in language. It’s also a high-stakes gambit. Because the obvious but unfortunate corollary is that if the case for real language skill is not persuasive, then the case for humane treatment fails with it, an outcome doubly tragic for Kanzi who spent his entire life in captivity to help decide the matter.

Whatever one’s stance here on Kanzi, it clearly illustrates the elision of science and ethics. It highlights the appeal but also the challenges and potential risks associated with moving from descriptive science to normative science, from concerning ourselves with “*how things are”* to concerning ourselves also with “*how things ought to be.”* The danger, of course, is that our commitment to how we think things ought to be is likely to color how we then think things truly are: our ability to study and describe reality distorted by how we wish it were. Whatever the potential value of anthropomorphism, this is a serious potential pitfall.

It’s important to be clear that, in cautioning against normative science and the logic in it that might put Kanzi’s case for more compassionate and humane treatment on the block, or the same case for any other animal, there is absolutely no critique of their abilities, intelligence or inherent worth. On the contrary, we should celebrate the abilities of other species and accord them due compassion and protection – however, we should do that without requiring that their abilities also be our particular brand of ability. We should respect, support, protect and conserve them for their own sake and not for how much they remind us of ourselves, which is both anthropocentric and conceited, as though ours was the only kind of life worth valuing.

In fact, surely it is the more remarkable that bats navigate in the dark in the way that they do, with a sensory ability entirely foreign to us. Surely, it is awe-inspiring to think that they can see things much as we do – for they *can* also see – but that they can also effectively “see” acoustically, meaning that they also form auditory profiles of shapes and obstacles in the world around them. In essence, then, they know both what the landscapes they inhabit *look* like and also what they *sound* like, which is frankly a bit hard even to fathom. And surely that is the better part of the “grandeur in this view of life” to which Darwin so famously alluded.

## Conclusion

Seyfarth et al. (1980a) published a landmark paper in *Science*, reporting evidence interpreted as language-like semantic communication in a primate. It had a profound impact. It represented the best case for semantic communication in animals and became the textbook example for it, catalyzing an enduring quest for semantics, syntax and other high-level features of human language in the communication of primates and other species. Ultimately, the original findings were revised, the case for vervet semantics disproven, and with it also support for the notion even of functional referentiality as the much diluted version of representation to which the primate semantics agenda had previously retreated (Wheeler and Fischer, 2012). Nevertheless, the broader research program engendered by the vervet story continues apace (Searcy, 2019). Why and how the vervet research had such impact warrants sober reflection.

There are a host of possible reasons why the vervet alarm story may have been so impactful originally, and why its legacy remains so enduring, a few of which have been considered here. They include the role of a contemporaneous paradigm shift in research on human and animal behavior and psychology in the middle 20th century and the role of key influencers in that shift. Chomsky was one key influencer in the Cognitive Revolution, following his mini-revolution in linguistics with bold proposals about Universal Grammar and an innate cognitive module dedicated to language acquisition [the so-called Language Acquisition Device (LAD)], both posited as necessary to overcome what he took to be the fundamental unlearnability of language by children. Griffin and Marler were key influencers carrying the cognitivist baton in the field of animal communication, where they followed Chomsky’s lead in foregrounding human language as the benchmark for sophisticated cognition in animals and so used explicit comparisons to human language to frame comparative study of communication in other species.

The resulting cognitivist movement proved tremendously productive generally, including in comparative psychology and in research on animal communication. However, it has over time also confronted some of its own limits, as many of its founding findings and propositions have been eclipsed: Chomskyan Universal Grammar has been successively diluted and ultimately, largely abandoned; the need for an innate language acquisition module has been similarly obviated by research now demonstrating the fundamental learnability of language by children; modern cognitivism recapitulates core elements of behaviorism in associationist processes undergirding the contemporary focus on neural networks, connectionism, and deep learning; and recent reviews of primate communication now acknowledge it is not semantic nor meaningfully language-like. The disruptive influence of paradigm shifts can be extremely productive. However, invariably they also sow the seeds of their own succession. Paradigms change, that reality is built into the paradigm concept. The long history of reversals in variously embracing versus eschewing mentalistic constructs in scholarly discourse over the last few centuries should make us especially wary that the current cycle in this fashion is either the best or the last.

The original vervet alarm call story appealed during a particular cycle that spawned a thriving mentalist, cognitivist paradigm. That paradigm provided a welcoming intellectual climate prepared to receive the vervet findings. The work also had broader popular appeal, tapping an anthropomorphic instinct or habit, effectively pre-prepared to receive and reward reports of human-like traits in animals. Some researchers will bristle at the latter suggestion. They will reject the possibility that anthropomorphism ever influenced their own work, being acutely aware of its checkered reputation in scholarly circles if not aware of its possible unconscious influence on them nevertheless. After all, instinct and habit are difficult to control. That’s the point. They are habitual and instinctual precisely because they are not under explicit and reflective control: like other common biases we continue to wrestle with – gender bias, racial bias – that operate widely, unconsciously, unwittingly. We are often not even aware of their influence. And even when we are aware, they can remain (maddeningly) immune to our formal, conscious and explicit attempts to mitigate and eliminate their influence.

For some, this history and its outcomes will give pause. That the original claims of semanticity in vervets have been recanted, and with them also broader claims of semantic communication in primates, may be troubling, possibly creating doubts about much else besides. Others may not be so affected, confident in the value of the much wider program of research on language parallels in animals that the vervet work helped to catalyze. There are many possible conclusions that could be drawn, and no doubt many possible opinions about the importance of other factors at play – formal intellectual paradigm shifts, spurred by key influencers, intersecting other universal human biases. But whatever one’s predilection on these matters, perhaps there can, at the least, be common commitment to the impartial and timeless prescription of Laplace, Williams and Sagan that our production and reception of research maintain a healthy circumspection; that we value and expect high standards of evidence, perhaps particularly for claims of an extraordinary nature.

## Author Contributions

The author confirms being the sole contributor of this work and has approved it for publication.

## Conflict of Interest

The author declares that the research was conducted in the absence of any commercial or financial relationships that could be construed as a potential conflict of interest.
